# Precision Nomothetic Medicine in Depression Research: A New Depression Model, and New Endophenotype Classes and Pathway Phenotypes, and A Digital Self

**DOI:** 10.3390/jpm12030403

**Published:** 2022-03-05

**Authors:** Michael Maes

**Affiliations:** 1 Department of Psychiatry, Faculty of Medicine, Chulalongkorn University, Bangkok 10330, Thailand; dr.michaelmaes@hotmail.com; 2 Department of Psychiatry, Medical University of Plovdiv, 4002 Plovdiv, Bulgaria; 3 IMPACT Strategic Research Center, Barwon Health, Deakin University, Geelong, VIC 3220, Australia

**Keywords:** depression, neuro-immune, oxidative and nitrosative stress, inflammation, mood disorders, biomarkers

## Abstract

Machine learning approaches, such as soft independent modeling of class analogy (SIMCA) and pathway analysis, were introduced in depression research in the 1990s (Maes et al.) to construct neuroimmune endophenotype classes. The goal of this paper is to examine the promise of precision psychiatry to use information about a depressed person’s own pan-omics, environmental, and lifestyle data, or to tailor preventative measures and medical treatments to endophenotype subgroups of depressed patients in order to achieve the best clinical outcome for each individual. Three steps are emerging in precision medicine: (1) the optimization and refining of classical models and constructing digital twins; (2) the use of precision medicine to construct endophenotype classes and pathway phenotypes, and (3) constructing a digital self of each patient. The root cause of why precision psychiatry cannot develop into true sciences is that there is no correct (cross-validated and reliable) model of clinical depression as a serious medical disorder discriminating it from a normal emotional distress response including sadness, grief and demoralization. Here, we explain how we used (un)supervised machine learning such as partial least squares path analysis, SIMCA and factor analysis to construct (a) a new precision depression model; (b) a new endophenotype class, namely major dysmood disorder (MDMD), which is a nosological class defined by severe symptoms and neuro-oxidative toxicity; and a new pathway phenotype, namely the reoccurrence of illness (ROI) index, which is a latent vector extracted from staging characteristics (number of depression and manic episodes and suicide attempts), and (c) an ideocratic profile with personalized scores based on all MDMD features.

## 1. Introduction

“Precision psychiatry” is a new hot topic in psychiatry. In his 2015 State of the Union address, US President Barack Obama announced his intention to invest USD 215 million in the National Institutes of Health’s “Precision Medicine Initiative”, and this initiative was renamed “All of Us” in 2016 [1,2]. Precision medicine is a medical model that advocates for customized healthcare by tailoring medical decisions, practices, and treatments to a subgroup of patients rather than a one-drug-fits-all approach [1]. Precision medicine refers to the classification of individuals into endophenotype classes based on their susceptibility to a particular disease, the biology of the disease they may develop, the response to a particular treatment, prognosis, and the process of tailoring medical treatments to each patient’s unique features [1,2,3,4,5,6,7].

In precision medicine, diagnostic testing is routinely utilized to choose appropriate and optimal treatments based on molecular pathological epidemiology and precision medicine biomarkers, namely the patient’s genetic makeup, and molecular or cellular data [1,3,4,5,6,7]. When pan-omics data are combined with the patient’s environmental and lifestyle data, a significantly greater level of predictivity is expected than when single indicator data are used [6]. These data enable the development of new endophenotype classes that can be used to make more targeted decisions about disease staging and prognosis, as well as the development of biosignatures (based on molecular biomarkers) that can provide an early warning of impending disease states even before they manifest. As such, precision medicine enables the development of novel preventive and therapeutic interventions tailored to endophenotypic subgroups or biosignatures, thereby increasing the cost-effectiveness of treatments and avoiding adverse side effects [1,3,4,5,6,7].

Precision psychiatry is now frequently described in psychiatric journals as a novel technique that has the potential to significantly advance psychiatric clinical practice, just as precision cardiology and oncology have been demonstrated to improve the treatment of cardiovascular disease and cancers, respectively [8,9,10,11,12,13].

The aim of this opinion paper is to examine precision psychiatry’s promise in using information about a depressed person’s own genes, pan-omics data, and environmental and lifestyle data to prevent, diagnose, or treat major depressive disorder (MDD) or a major depressive episode in bipolar disorder (MDE), or to tailor preventive measures and medical treatments to endophenotype classes of depressed patients in order to achieve the best clinical outcome for each individual.

## 2. Methods

In this conceptual analysis, I will review (1) the aims of precision medicine models and the three steps that characterize precision medicine; (2) the current misconceptions of precision medicine models held by psychiatrists; (3) the core issues in psychiatry which prevent the development of precision psychiatry models of MDD/MDE; and (4) how to construct new precision nomothetic models in mood disorders.

## 3. The Aims of Precision Medicine and Machine Learning

**Figure 1:**
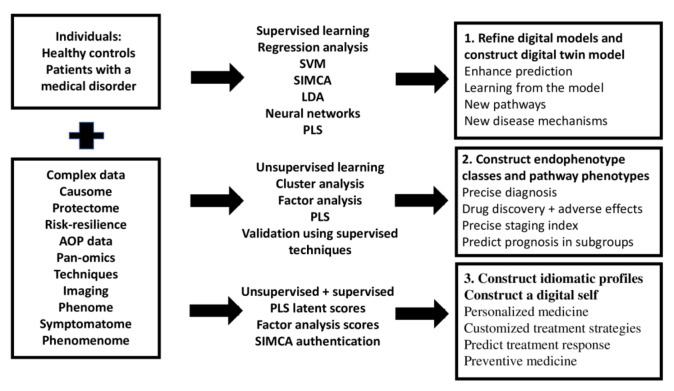
The three different steps which characterize precision medicine, the machine learning techniques that can be used to achieve these goals, and the products of these techniques. AOP: adverse outcome pathways; SVM: support vector machine, SIMCA: soft independent modeling of class analogy; LDA: linear discriminant analysis, PLS: partial least squares path analysis.

The foundation for precision medicine is built on the amassment of complex and big datasets (pan-omics, electronic registries, biobanks, complex imaging data), as well as the application of machine learning techniques to learn from the data and improve predictions of outcome variables (classifications, disabilities, prognosis, course of disease, treatment response, adverse drug reactions) at the disease, endophenotypic and personal level [6,7]. Three steps are emerging in the precision medicine approach, namely: (1) the optimization and refinement of classical disease models, (2) the precision medicine approach to construct endophenotype classes and pathway phenotypes or biosignatures, and (3) the personalized medicine approach, which focuses on a particular patient. Figure 1 shows these three different steps which characterize precision medicine, the machine learning techniques that can be used to achieve the three goals, and the products of these techniques.

### 3.1. Step 1: Optimize Existing Models

First, the application of complex and large pan-omics datasets and supervised machine learning approaches allows for the improvement of the prediction of classical disease models (of diagnosis, prognosis, and drug discovery) and the optimization of the criteria of existing diagnostic classes [14,15,16,17,18]. Moreover, some of these supervised methods allow us to learn from the data and may reveal new pathways underpinning the disease. These techniques may be used to construct data-driven models that can be employed to predict class membership—for example, who is classified as an MDD/MDE patient or who is classified as a control. When such data have a cross-validated accuracy of more than 90.0%, the prediction model can be used to predict class membership [14], to optimize existing models, learn from the data, and classify individuals into distinct categories. Table 1 shows the definition and aims of supervised pattern recognition methods as well as examples of major supervised techniques.

The labeled data to conduct supervised pattern recognition methods always consist of paired input (explanatory, independent variables, e.g., features of MDD/MDE) and correct output (dependent) data, namely the predefined classes (e.g., a correct depression model of MDD/MDE versus controls) or continuous outcome scores, for example, severity of illness. When no correct output data are available, supervised learning cannot be employed.

Support vector machine (SVM), linear discriminant analysis (LDA), neural networks, independent modelling of class analogy (SIMCA), logistic and multivariate regression, and partial least squares (PLS) path modelling are some of the supervised machine learning approaches that can be employed to achieve this goal, and they all significantly enhance prediction accuracy either from prespecified classes (SVM, LDA, neural networks, SIMCA, and logistic regression) or quantitative scores (multivariate regression) [18,19,20]. A combination of these methods is recommended because some are better suited for classification (e.g., SVM and neural networks), the prediction of quantitative outcome data with (pathway analysis) and without (multiple regression analysis) mediated effects, and learning from the data (SIMCA, neural networks, multivariate or logistic regression analysis), while other methods (e.g., SIMCA) are better suited for authenticating cases as healthy volunteers or as patients [14,21]. Some of these approaches and their applications in precision medicine will be discussed in Section 4.

The results of these supervised machine learning techniques (top-down with a priori definition of a class) should be employed to constantly update the original disease model through deductive reasoning—namely, the original model laws are coupled with the conclusions of machine learning to refine the original model. For example, using precision medicine in conjunction with machine learning can improve the detection and diagnosis of cardiovascular disorders, as well as the promotion of cardiovascular health and the reduction in cardiovascular risk [6]. A combination of different MRI techniques in precision models (including gadolinium enhanced MRI and diffusion weighted imaging) is now used to improve the diagnosis of brain tumors [22,23].

### 3.2. Step 2: Uncover Endophenotype Classes and Pathway Phenotypes

Second, through the use of machine learning, multi-modal data (including pan-omics data, symptoms, health-related quality of life (HR-QoL) and disability scores) can be combined to construct endophenotype classes and pathway phenotypes that are relevant regarding specific pathophysiological events, treatments, staging, prognosis, and adverse effects of medications [1,3,4,5,6,7]. For instance, not only various MRI methods but also morphological measurements of imaging abnormalities and tumor biomarker testing may help to define endophenotype classes of individuals with brain tumors (glioblastoma) who may react differently to novel therapies or have a varied prognosis [22,23,24].

An endeavor such as this should be based primarily on unsupervised learning (no a priori definition of a class or model) with the assumption that, within a correct disease model, new clusters of patients (endophenotype classes) or new constructs that combine symptoms and biomarker data (pathway phenotypes or biosignatures) may be uncovered. Table 1 shows the definition and aims of unsupervised pattern recognition methods as well as examples of the major unsupervised techniques. For instance, several clustering approaches (Forgy’s method, K-means, K-median, and hierarchical clustering) may be used to identify novel endophenotype classes (depression subgroups) within the depressed population, while factor analysis or PLA path analysis may be used to uncover new pathway phenotypes [25,26].

Pathway phenotypes are different from endophenotype classes in that they are based on quantitative scores of interrelated symptoms, biomarkers, or genetic or environmental features of depression [14,27]. Principal component analysis (PCA) and factor analysis are adequate tools to detect pathway phenotypes. The prerequisite is that strongly interrelated features belong to the same reflective latent vector, which in fact is the cause of all its manifestations [18,19,20]. For example, the abstract construct “HR-QoL” may be conceptualized as a reflective latent vector (factor) extracted from self-rated quality of life domain scores, such that the latent vector HR-QoL is the cause of all its manifestations [28]. If this factor also comprises biomarkers or genetic data, a pathway phenotype is formed, and the latent vector scores may be used as quantitative severity scores of this biosignature [19]. Of course, the factors should comply with stringent quality criteria as explained in Almulla et al. [18] and Section 4.

### 3.3. Step 3: Towards the Personalized Approach

The first and second applications of precision medicine allow us to achieve the ultimate goal of precision medicine, namely personalized medicine. For example, in precision cardiology, machine learning in conjunction with pan-omics and wearable electronic devices which monitor cardiovascular functions, coupled with lifestyle and environmental factors, may aid in developing preventive treatments tailored to individual patients or individuals at risk of developing atherosclerosis [6]. In precision oncology, the clinical utility of this approach has been demonstrated by genotyping tumor tissues from cancer patients, which may provide information on the type of drugs to be used and the drugs with the fewest side effects [29].

What is critical is that precision medicine should begin by refining the existing illness model (step 1) and then delineating new endophenotype classes and pathway phenotypes (step 2) that can be utilized at the personalized level (step 3) (see Figure 1). Consequently, endophenotype classes, pathway phenotype scores, and scores on related markers and feature sets may be utilized to generate digital models of patients and controls as well as an idiomatic profile for each case (a digital self for each individual). As discussed in Section 5 and Section 7, PLS path analysis, SIMCA, and factor analysis are well-suited to this personalized approach. For example, PLS analysis allows us to compute feature set scores that are consequently used to construct digital models and digital selves, while SIMCA is used to compare the idiomatic profile of an individual (the digital self) with the digital models (in statistical terms: to project a case within the class model limits of a diagnostic group).

Once a digital model of healthy controls is built (e.g., using SIMCA), this model enables us to delineate normal functioning (a case may be authenticated as a healthy human or not) and optimization and maintenance of performance (some features of a normal control may deviate from the norm). Once a SIMCA digital model of depressed cases based on a newly developed endophenotype class is formed, the latter can be used to diagnose malfunctioning (who suffers from severe depression), predict malfunctioning (making inferences about who may later develop severe depression), prevent malfunctioning (prevention in high-risk subjects), repair and restore (best treatment for severe depression), and examine functionality following repair (treatment response versus partial or non-response).

Before I describe how we utilized SIMCA, PCA, PLS-path and clustering analysis to create digital models and digital selves for patients and controls [14,15,16,17,19,20,21,26,30], I will first provide some context for those approaches (see Section 4). While these approaches are the cornerstone of our precision psychiatry approach, we always combined them with additional machine learning techniques such as SVM, neural networks, PCA plot, correspondence factor analysis, and regression analysis, as previously mentioned [14,15,16,17,18].

## 4. Specific Supervised and Unsupervised Pattern Recognition Methods

### 4.1. SIMCA: A Supervised Learning Method

SIMCA is a class modeling strategy that uses PCA to model patient and control groups based on the relevant features including genetic and environmental data, pan-omics and other data [14,21]. SIMCA is a supervised learning approach that starts with labeled datasets, namely paired input data (all disease features) and correct output data (predefined categories) [21,30,31,32]. SIMCA generates separate principal component analysis (PCA) models for each class (e.g., depressed patients and healthy controls), using a cross-validation technique to determine the number of PCs, which may vary by class. Both study samples are divided into a training or calibration sample (e.g., 50% of patients and 50% of controls) and a validation sample (e.g., 50% of patients and 50% of controls) (also 50%) to cross-validate the results.

**Figure 2:**
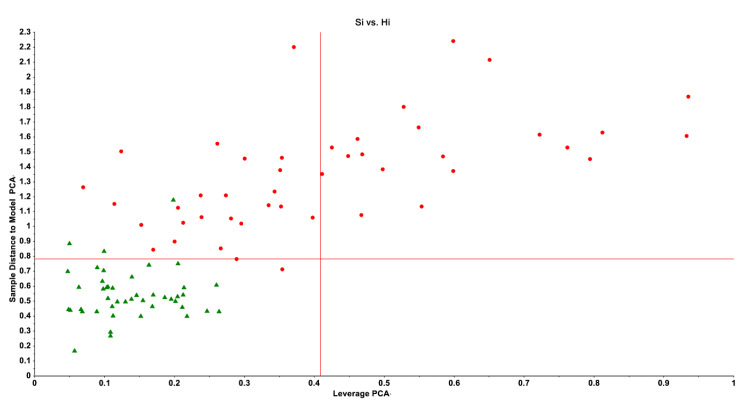
A Si/Hi plot (distance between the cases and the class model/distance between the cases and the class center or leverage) obtained by soft independent modeling of class analogy (SIMCA). Green triangles: normal volunteers; red bullets: patients with major depression.

SIMCA generates two distances, namely the distance between the cases and the class model (Si) and the distance between the cases and the class center (Hi or leverage), which are used to define the class limits (borders) and decide whether subjects should be allocated to or rejected from a class. These calculated distances are utilized to generate two-dimensional graphs, including a Si/Hi plot, which depicts the subjects’ relative distance to the class model and the distance to the class center. Figure 2 illustrates such a Si/Hi plot, in which we build a PC model surrounding healthy controls and project severe depression cases into the SIMCA model’s class limits.

The Si and Hi distances define the class limits (borders) of a diagnostic class (e.g., the lower left quadrant which delineates the limits of the normal control class). Thus, all normal controls are allocated to the normal control class, although there are three outliers (which decrease the specificity of the model). Most depressed patients are not authenticated as normal controls, except two cases that should be considered as intruders.

Thus, controls may be allocated to their target class (in Figure 2, the normal control class) based on their estimated critical class boundaries, and so establishing their authenticity as normal controls. Additionally, cases with a clinical diagnosis of depression but who intrude into the target class are in fact incorrectly diagnosed as patients, as they appear to have similar features to normal controls. Additionally, if cases fall into both categories, the model should be adjusted for improved performance by selecting more specific features. In Figure 2, just two depressive cases are intruders, indicating a reasonable sensitivity of the predictive model’s accuracy [14,30].

Additionally, SIMCA generates two figures of merit: (a) the confusion matrix, which contains information about classification accuracy, sensitivity, and specificity; and (b) the model-to-model distance, with a distance greater than 3 indicating significant differences between groups and distances greater than 20–30 indicating qualitative differences between predefined classes [14,15,30]. Furthermore, SIMCA computes a discrimination plot that illustrates the discriminatory power of all input features in distinguishing patients from controls. As such, SIMCA is a powerful approach in Step 1 because this method allows us to optimize disease models and predict class memberships, and in Step 3 because it enables us to compute SIMCA models of controls and patients in which the projection of unknown cases may allow us to authenticate those unknown cases.

### 4.2. PLS Path Modeling: A Supervised Technique

PLS modeling enables the following: (a) the construction of causal models linking genetic factors to biomarker pathways, cognitive dysfunctions, staging characteristics, and symptoms of depression; (b) the construction and inclusion in the model of latent vectors that are based on variables that cannot be measured directly (unobservable variables), such as HR-QoL; (c) the delineation of mediation effects, such as the effects of immune genes or environmental factors on HR-QoL which are mediated by neuro-immune pathways; and (d) the estimation of complex multistep mediation models that link numerous single indicator variables (e.g., age or sex) and latent vectors (e.g., HR-Qol) in structural causal paths without imposing distributional assumptions on the data [16,19,25,26,33,34].

Different indicators and latent vectors extracted from a set of strongly interrelated indicators can be entered as input variables predicting the outcome variable [25,26]. Only when the quality of the model meets predefined quality requirements is a complete PLS analysis with 5000 bootstrap samples conducted. The most important requirements are that the model’s overall quality is appropriate, that the latent vectors have adequate composite reliability, that their average variance extracted is greater than 0.500, and that all indicators of the latent constructs have a high factor load [14]. Complete PLS path analysis with bootstrapping (5000 subsamples) is then used to compute path coefficients with exact *p*-values, and total, specific indirect and total indirect effects, which assess the significance of mediated paths. Moreover, once the model has been validated, latent variable scores of the different indicators and latent constructs may be computed, yielding a reduced number of feature set scores, reflecting the impact of various genetic, environmental, pathway, cognitive and symptom data.

As a result, PLS path modeling is a powerful method in Step 1 because it allows for the construction of new models or the optimization of classic models of complex disorders, which are characterized by causal links from genetic and environmental factors to pathways and severity of depression. It is also a powerful method in Step 2 because it allows us to build pathway phenotypes by combining strongly interrelated biomarker pathways and staging data in validated latent constructs that may predict the outcome. Furthermore, PLS path modeling is a powerful method in Step 3 because it allows one to compute personalized scores that reflect all features of an illness, thereby shaping a personalized idiomatic profile or digital self.

### 4.3. Principal Component Analysis: An Unsupervised Machine Learning

Joint principal component analysis (PCA) is performed on the features of depressed cases with or without controls using a standard deviation weighting procedure and a 20-fold cross-validation scheme in order to visualize the distribution of the cases in a 2D space [14,18,30]. The distributions of both classes are shown by examining various combinations of PCs, with PC1 versus PC2 being the most important since it includes most of the variance in the data. When clusters of patients with clear boundaries (a large street) are discovered, it suggests the presence of endophenotype classes. The correlation loading plot enhances the comprehension of this PC plot by displaying the correlation loadings for all variables in PC1 and PC2 with respect to two ellipses, with the outer ellipse representing 100% explained variance and the inner ellipse representing 50% explained variance. PC plots are an effective tool for illustrating the data distribution in Step 2, since they depict the data distribution [18].

In Section 4.2, I discussed how to create pathway phenotypes using factor analysis as part of PLS path modeling. However, such investigations should be preceded by exploratory factor analysis (EFA), a data-driven technique for determining the factor structure of associated indicator variables [18]. Thus, by combining biomarker pathways and clinical data into a single component, this technique allows for the building of a pathway phenotype if one general component accounts for a considerable percentage of the variance (>50%) and has high loadings for all indicators (>0.6). Additionally, the number of retained factors should be one, as determined by the Hull test, parallel analysis, or Schwartz’s Bayesian information criterion; and this factor should be unidimensional, as determined by tests for closeness to unidimensionality, such as the mean of item residual absolute loadings, unidimensional congruence, and explained common variance [18]. Apart from discovering new patterns in a dataset, this method is also used as a feature reduction method whereby a larger number of interrelated indicators are reduced to one factor. Finally, using this method, factor scores may be computed that reflect the severity of the constructed pathway phenotypes.

### 4.4. Clustering Analysis: An Unsupervised Learning Technique

Clustering analysis is an unsupervised machine learning approach that may be used to discover hidden patterns or clusters of cases within a population sample based on common attributes or communalities. Clustering is an unsupervised technique, since it makes use of unlabeled and uncategorized data without predefining any classes [21]. Table 1 summarizes some of the most frequently used clustering methods. Clustering is a powerful method in Step 2 because it allows one to discover new endophenotype classes when applied to the features of depression. Consequently, the characteristics of the clustering-derived classes may be investigated using basic descriptive statistics, analysis of variance, and contingency analysis and, consequently, the clustering solution and model algorithms should be confirmed in independent subsamples, for example, using SVM, SIMCA or neural networks, which enable the estimation of the cross-validated accuracy in an output sample [14,21]. When these subclasses are thoroughly cross-validated and include genetic or environmental data, pathway biomarkers, symptoms, and/or cognitive scores, a new endophenotype class is formed [14].

## 5. Core issues in Psychiatry Which Prevent the Development of Precision Depression Models

### 5.1. Contemporary Depression Research Is a Chaos of Many Concepts, Diagnoses, and Labels

It is a noble goal to make psychiatry more precise, but psychiatry is the most imprecise medical discipline that exists. Furthermore, it is not only imprecise, but also a jumble of various model concepts, conceptual frameworks, and classifications of the same disorder. There is no other medical discipline that offers multiple model versions of a disease. However, in psychiatry, this is not only common, but it is an art to generate new classification models, either through consensus meetings or just by publishing a newly created model. Thus, according to Zachar and Kendler [35,36], major psychiatric illness may be conceptualized using a variety of concept models, including organic disease models, altered function models, biopsychosocial models, harmful dysfunction models, dimensional models, alternative models, and interpersonal models. These models are proposed even without any evidence. For example, according to the interpersonal models, disturbed relationships are the cause of severe psychiatric illness, although no evidence for this has ever been provided. Nonetheless, new interpersonal treatments are frequently advocated and used based on this untested and unfalsifiable theory. Passing Karl Popper’s falsification criteria [37] is something far off for most psychiatrists. Psychiatry is also the only discipline that proposes a non-model in which a narrative account may reveal the true cause of a major psychiatric illness, including MDD/MDE [35]. Producing a non-model of a severe medical disease, e.g., atherosclerosis, would be absurd, but not in the field of psychiatry.

**Figure 3:**
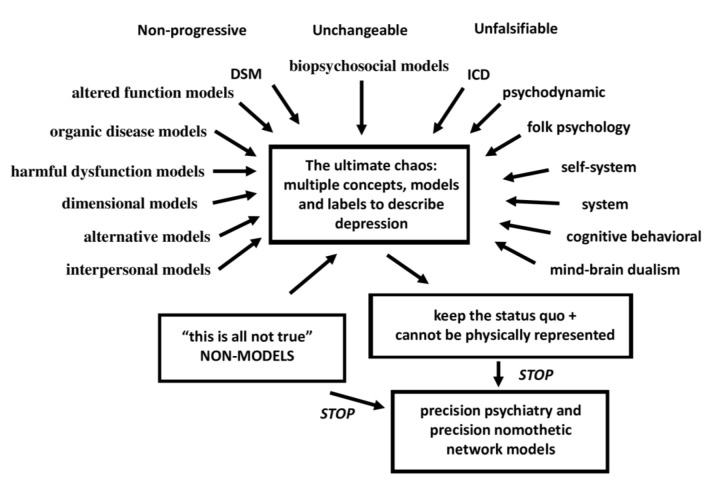
The status quo chaos and non-models of psychiatric depression concepts.

Previously, we discussed the many competing and even mutually antagonistic approaches to understand depression, including many non-falsifiable theories including psychoanalysis (depression is a defense against loss and mourning), psychodynamic psychiatry (depression is the result of a pathological object relationship between parts of the self), commonsense or folk psychology (depression is a response to a psychological problem), self-system theory (depression is a response to a self-problem), systemic therapy (depression is caused by systems such as the family), cognitive-behavioral therapy (depression is the result of negative thoughts), and the mind–brain dualism (neural and/or mental processes interact to cause depression) [38]. Critical psychiatry with psychiatric survivor networks, a recent development indicating that contemporary psychiatry is in deep crisis, calls into question the decontextualization of experience, scientific methods, knowledge base, psychiatric practice, and treatment, and accuses status quo psychiatrists of harmful and unethical principles [38]. Figure 3 depicts this status quo chaos and the non-models in psychiatric depression concepts.

This extreme chaos in modern status-quo psychiatry is further demonstrated by the numerous proposed MDD/MDE subtypes, subclasses and labels and the constant establishment of new depression subtypes with new case definitions that may vanish after a while [38]. Psychiatric epidemiologists do not contribute to the resolution of the overall chaos; in fact, they exacerbate it. For example, the existence of distinct diagnostic categories is conceptualized without any cross-validation, and then it is demonstrated that they all exhibit significant comorbidity. This, in turn, is followed by a large series of pointless papers discussing the prevalence and incidence of the disorders with and without comorbidities, the effects of various threshold values, and whether these classes are distinct or shape a continuum.

Thus, any psychiatrist can invent new models, diagnostic criteria, diagnostic labels, and therapies, and new comorbidities, all of which are then published in high-impact psychiatric publications as if they were high science. However, the newly introduced classes and new case definitions are based on unverified ideas of some highly or less highly cited psychiatrists and are sometimes shaped through consensus discussions but never validated or cross-validated with accurate machine learning models [8,38,39].

Mental health professionals have typically relied on psychosocial or mind–brain theories to explain mood disorders, including MDD/MDE. An internationally recognized psychiatric superstar such as Kendler [40] asserts that the goal should be to understand how the psychosocial environment interacts with networks inside the mind–brain system to create mental diseases and asserts that the conceptual foundation for psychiatry should be based on the acknowledgment of a bidirectional causality between the brain and the mind as well as between the mind and the brain. However, these models have proven unsatisfactory because we constructed new precision psychiatric models of MDD/MDE based on adverse outcome pathways (AOP) in peripheral blood, which are activated by interactions between genetic and environmental factors, including psychological trauma and increased bacterial load in the peripheral blood (see below) [25,26]. As such, psychiatry is the only medical discipline that does not adhere to the premise that medical concepts should pass the critical rationalism tests as proposed by Karl Popper [37] namely that models should be falsifiable, changeable, parsimonious, and progressive. As a result, contemporary psychiatry is perpetually in chaos without a compass to guide it.

### 5.2. The Core Issue Which Undermines Progress in Precision Depression Research

Even before precision psychiatry could start, it was criticized due to the fact that its foundations are outdated and flawed because one cannot reduce mental suffering to biological essences [41,42]. According to the latter authors, precision psychiatry does not constitute an adequate replacement to current phenomenological classification systems, since biological processes do not capture qualia and meaning, mental suffering or even pathology. Among the main criticisms is that mental events are not physically represented, and thus finding biomarkers is not important to understanding mental processes. Phrased differently, for these authors falsifiability is not even a concern because their dogma is that the mind cannot be falsified. The authors then give falling in love as an example: falling in love would be a “boundary experience” that can bring some people dangerously close to experiencing intense mental suffering with a loss of ground and autonomy that may manifest as a mini-neurosis, a mini-psychosis, or a behavioral pathology [41]. Such a view is also supported by sociologists who consider that “psychiatry transformed normal sorrow into depressive disorder” [43] and by psychiatrists who caution about the medicalization of normal human behavior and the emotional responses of normal life such as sadness, low mood, feeling blue, grief and demoralization [44,45].

Unconsciously, these authors have addressed the root cause of why psychiatry and precision psychiatry cannot develop into true sciences, namely that there are no reliable and validated “laws” or correct models to discriminate a normal emotional response with depressive feelings from their pathological counterpart, including MDD/MDE, conceptualized as a severe medical illness or a major psychosis, and labeled as “Major DysMood Disorder” (MDMD) [25,26]. As a comparison, is there really a spectrum between, for example, a tension headache and a headache caused by a brain tumor? By analogy, is there really a spectrum from an emotional response or a process in the mind (e.g., being in a mini-neurosis due to falling in love or the consequent sadness after breaking up) and MDMD symptoms such as feelings of guilt and worthlessness, profound despondency and despair, self-blame, anhedonia, emptiness, hypoesthesia, psychomotor retardation and agitation, diurnal variation, neurocognitive deficits, severe physio-somatic symptoms, inexplicable desperateness, and (mood-congruent) delusions or hallucinations [38]. Thus, the medical approach promoted in the current paper does not aim to medicalize normal human responses but rather seeks to discriminate a normal human response from a severe medical illness. In fact, the main aim in psychiatry should be to discriminate MDMD from a human emotional distress response, just as it is important to diagnose a headache due to a brain tumor and differentiate it from a tension headache. The mainstream view of psychologists, psychotherapists, sociologists, psychosocial psychiatrists, and psychiatric epidemiologists that MDD/MDE is an expression of a dysfunction in the mind–brain axis and that there is a spectrum from a distress response to MDD/MDE [38] may be quite unethical if one fails to recognize MDMD as a major psychosis or a serious medical illness.

Thus, the critical issue is that psychiatry currently lacks a correct disease model for MDMD. Rather than changing unproven case definitions of depression with additional unproven criteria, psychiatry should focus on establishing innovative validated or cross-validated models of MDMD. Indeed, there is currently no evidence base for precision psychiatry because there is not even a correct disease model of depression. Without a correct model, supervised machine learning cannot be applied and, as shown in Section 3 and Figure 1, the prerequisite for Steps 2 and 3 is a cross-validated correct model.

### 5.3. The Gold-Standard Psychiatric Rating Instruments Are Impediments

The gold standard DSM [46] and ICD [47] categorization and case definitions of MDD/MDE are unreliable and show often a flawed intraclass kappa reliability with little agreement among psychiatrists [38,48]. More importantly, these categories do not allow one to distinguish between individuals who suffer from an emotional response with depressive symptoms, minor depression, MDD/MDE and those who suffer from MDMD. A direct consequence is that psychiatrists use rating scales to assess depression, whereby a small increase is then regarded as reflecting depressive symptoms, MDD/MDE, or even a severe pathological condition. Never mind that these rating scales have virtually no validity because: (a) the most common depression scales do not comply with the unidimensionality criterion and measurement invariance; and (b) the items of the scales are derived from folk psychology (“I believe I am depressed”) which are, after some window dressing, translated into statistical entities that are used as proxies for a severe medical disorders such as MDMD [38,48].

The most disturbing aspect is that all the concepts and models described in Section 5.1 and Section 5.2. and the gold-standard case definitions of the APA and WHO (all except the non-models) are based on a dogma-like, top-down-generated method [38,39,48]. Indeed, the DSM and ICD diagnostic classification systems treat the most fundamental components of any medical disease as auxiliary information that may or may not support their new dogma-like constructs, including the causome/protectome, AOP (multi-omics data), and brain circuitry assessments [38,39,48]. Worse yet, these flawed methodologies preclude falsification and restrict deductive and inductive causal reasoning because no general conclusions are allowed to be drawn from the most important features of MDMD [38,39,48]. Notably, the DSM/ICD models fail Karl Popper’s tests of critical rationalism, as they are not only unfalsifiable (top-down approach and no refutation is allowed), but also unchangeable (ex-consensus-based committees of professionals make the criteria) and non-progressive (because they do not consider the state-of-the art information) [48].

Following the development of unfalsifiable mind–brain models and non-validated case definitions of MDD/MDE, it is absurd that the same authors and the American Psychiatric Association then declare that decades of research in the field of biomarkers, brain circuits, and genes has failed to produce any biological subgroup or biomarkers of major psychoses, and that there are no valid, and replicable associations between biology and mental suffering [35,36,46,49]. In fact, the lack of a cross-validated, correct phenotype (a correct depression model) precludes the use of supervised learning technique in Steps 1, 2 and 3 of the precision medicine process and impedes the normal development of precision psychiatry. As such, it may appear as though the American Psychiatric Association, psychotherapists, social psychiatrists, psychologists, psychotherapists, and psychiatric epidemiologists are attempting to defend phenotype errors and prevent changing the status quo, thereby cementing their position as the most critical components of psychiatry (see Figure 2).

## 6. Precision Medicine in Psychiatric Research

Examining recent papers published by psychiatrists that discuss precision medicine and its application in psychiatry reveals that many authors do not recognize the need for a correct model before machine learning can be applied.

Insel [50] described the Research Domain Criteria (RDOC) project of the NIMH as the precision medicine for psychiatry. RDoC relies on dimensions of psychopathology as critical measures, whereby psychopathology is caused by aberrations in neural circuits in the brain and should be examined using a matrix with eight columns, namely genes, molecules, cells, circuits, physiology, behavior, self-reports, and paradigms, and a number of rows, including memory, rewards, threats, and perception. Nonetheless, this is again a top-down dogma-like construct whereby the NIHM dictates some rows and columns but does not provide any evidence of how this matrix was constructed and why some factors are in the rows and not in the columns and vice versa. Put differently, there is no evidence-base for the top-down dogma-like RDoC matrix approach. In addition, the number of combinations in this matrix is limited and does not allow the unsupervised discovery of new endophenotype classes or pathway phenotypes.

Zanardi et al. [13] published a paper on precision psychiatry in clinical practice, defining it as “a method for integrating biological and environmental information in order to personalize treatments and complement clinical judgment” and claiming that it represents a revolution in psychiatric care. For Passos et al. [11], precision psychiatry encompasses “big data and machine learning to move beyond evidence-based group-level approaches into individualized care”. They claim that the next step in precision psychiatry is “to incorporate tools from machine learning guided trials for individualized interventions, providing a new generation of findings in psychiatry, beyond current group-based approaches.”

Salazar de Pablo et al. [12] propose that implementing precision psychiatry may help to predict diagnostic models (namely psychiatric disorders versus controls), prognosis (in terms of disabilities), and treatment response (in psychiatry but especially in psychosis) at the individual level. However, this is a systematic review of validated supervised prediction models, which are not examples of precision psychiatry, because the latter primarily aims to develop endophenotype classes derived from unsupervised learning experiments.

Arns et al. [51] describe stratified psychiatry as tomorrow’s precision psychiatry. These authors argue that stratified psychiatry is a way of subgrouping depressed patients with similar biomarker profiles to enhance the probability of a clinical response to on-label antidepressants. For example, this approach would allow for tailoring on-label treatments to a specific EEG profile, thereby maximizing the clinical efficacy of selective 5-HT reuptake inhibitors and not selective noradrenaline reuptake inhibitors. Nevertheless, the major problem with this approach is that a) biomarkers are found in networks of highly intercorrelated gene expression, protein–protein interactions, and multi-omics network data that interact with neurocognitive functions and the brain connectome data, making stratifying one or another biomarker or a group of biomarkers meaningless [52]. Most importantly, as explained in Section 4.2, a correct depression model should be based on the causal links between the building blocks of an illness (see also below).

Fernandez et al. [9] gave another definition of precision psychiatry: “Precision psychiatry aims to transform the psychiatric landscape through a bottom-up approach applied to pan-omics using system biology and computer science to compute a biosignature, which in turn may be used in a top-down approach to help to understand domains that differ from components but allow to construct endophenotypes” [48]. In another paper, the authors employ what they believe to be precision medicine, but instead demonstrate the outcomes of a top-down experiment utilizing LDA (not the most appropriate supervised classification technique) to differentiate patients diagnosed with inaccurate DSM case definitions from controls [53]. Unsurprisingly, combining immune-inflammatory biomarkers with cognition tests results in an inaccurate confusion matrix, with sensitivities ranging from 80% to 84% and specificities ranging from 71% to 73%. The authors then assert that a ROC curve of at least 70% is a success, despite the fact that such figures of merit do not allow for a precise prediction, as this requires a cross-validated accuracy of at least 90% [14]. Their findings indicate that while there are some variations between the groups, their technique cannot be described as precision psychiatry because the authors did not even complete Steps 1 and 2 (and 3) to delineate a correct depression model and more precise endophenotype classes.

Two other descriptions come from the Max Planck Institute [10] and Bzdok and Meyer-Lindenberg [54]. The latter ascertain that “modern machine learning approaches may have a natural potential to improve the well-being of psychiatric patients.” To that end, machine learning could be used to make measurements of the brain, behavior, and genes, allowing us to objectively measure endophenotypes, which could allow for early disease detection, individualized treatment selection, and dosage adjustment to reduce disease burden. The Max Planck Fellows Group for Precision Psychiatry [10] proposes that machine learning may be used to extract patterns from complex databases that can be used to predict individual illness courses and outcomes rather than just differences between patient groups. Both groups emphasize the personalized medicine approach, in which machine learning is used to extract signatures from pan-data (clinical, neuropsychological, imaging-based, and genetic) that can be used to predict drug effects in individual patients, either unwanted or desired effects.

Nevertheless, all studies mentioned in this section miss the point that precision medicine is based on a correct outcome depression model, which unfortunately does not exist (Step 1), and then delineate new endophenotype classes and pathway phenotypes (Step 2) that can be used at the personalized level (step 3).

## 7. Precision Nomothetic Networks of Mood Disorders

Overall, it appears that current MDD/MDE concepts and research have not resulted in a “correct” disease model that is regarded as the only model by all psychiatrists. When cardiologists use the term “atherosclerosis,” everyone understands what they are talking about. However, all that psychiatrists have is a jumble of models, concepts, and labels, as well as gold-standard case definitions that are not valid but have cemented dogmas that preclude any adjustments based on deductive and inductive causal reasoning. Other psychiatrists then propose unfalsifiable models or even non-models of the same disorder. Others loudly proclaim precision psychiatry a pseudo-science even before it can demonstrate its merits, because the mind and qualia cannot be quantified. Models and case criteria never met or cannot meet Popper’s criteria, but all this is irrelevant to most psychiatrists. The root cause for the fact that psychiatry has not developed into a true science and that precision psychiatry models cannot be developed is that there are no correct models for discriminating between an emotional response such as sadness and its pathological counterpart, namely severe depression, as a major psychosis.

Since the 1990s, my laboratories have been using machine learning techniques such as SIMCA, PCA plots, and pathway analysis to delineate novel endophenotype classes using biomarker assays. For instance, we demonstrated, using both supervised (SIMCA) and unsupervised (PCA) machine learning, that MDD/MDE, particularly when melancholia features are present, are qualitatively distinct classes based on various T-cell activation markers and defined a subgroup of patients with a T-cell activation endophenotype [55,56]. We delineated a new MDD endophenotype class using cluster analysis which was validated by lowered levels of tryptophan, a neuro-immune biomarker [30].

Recently, we developed a new precision nomothetic medicine method in conjunction with machine learning methods, including SIMCA, PLS path, cluster and factor analysis which allowed us to (a) build validated disease models of depression; (b) discover a new endophenotype class, namely MDMD, and a new pathway phenotype, namely the reoccurrence of illness index (ROI); and (c) construct digital models of MDMD and controls and compute a digital self of depressed individuals [25,26].

### 7.1. Conceptual Framework and Validation of A New Precision Model of MDMD

**Figure 4:**
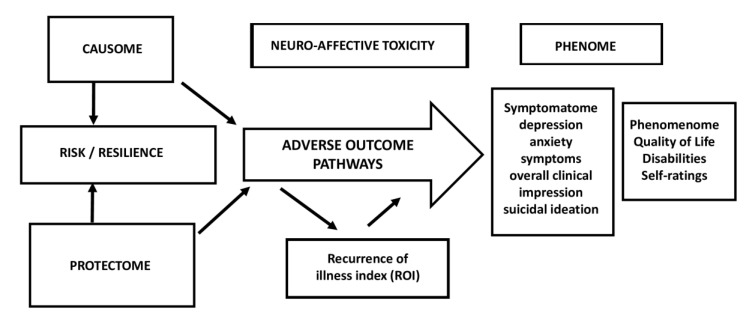
A knowledge-based causal framework of depression based on causal reasoning and current state-of-the-art research that describes the causal relationships between the various components of clinical depression.

The term “nomothetic” refers to the proclivity for deriving laws from indicator (independent) variables that account for the variability of phenomena and allow for model generalization, which is governed by mathematical laws that serve as explanations for the disease. Our precision nomothetic approach is based on elucidating the causal relationships between the most critical components of a complex psychiatric disease, such as depression. Table 2 and Figure 4 show the different building blocks of “depression” as a medical disorder.

First, causome (e.g., early lifetime trauma, genes including paraoxonase 1 (PON1), increased bacterial translocation) and protectome (genes, lifestyle factors including nutrition) features can be combined into a risk/resilience ratio via feature reduction, for example, by considering the patterns of interaction between genes and environmental factors (PON1 gene and ELT interaction [19,57].

Second, adverse-outcome pathways (AOPs), which include molecular, intracellular, neuro-immune, oxidative and nitrosative stress (O&NS), and metabolic pathways. The latter can be used to create constructs that reflect more specific pathways, for example, by combining O&NS biomarkers into a latent vector extracted from multiple O&NS indicators (feature reduction using PCA).

Third, brainome features, namely the totality of brain region abnormalities either structural or functional, including changes in the connectome [58].

Fourth, cognitome features, namely the aggregate of impairments in neurocognitive functions, including in attention, episodic and semantic memory, working memory and executive functions [57].

Fifth, and this is unique to mood disorders and other disorders with recurrent episodes (e.g., autoimmune disorders), the ROI [19,57]. In patients with MDD/MDE, we conceptualized the ROI as a latent vector extracted from the number of depressive and (hypo)manic episodes, the number of suicidal attempts and the lifetime history of suicidal ideation. In fact, the strong loading of all these indicators on the same ROI latent vector shows that MDD/MDE (and thus unipolar and bipolar depression) share a common core (the pathophysiology of ROI) that underpins the number of depression and (hypo)manic episodes and suicide attempts [25].

Sixth, the phenome consisting of the symptomatome and phenomenome. The symptomatome is the aggregate of the broad spectrum of symptoms that are characteristic of the illness, including severity of depression, severity of depressive symptoms such as feelings of guilt and worthlessness, profound despondency and despair, self-blame, anhedonia, emptiness, hypoesthesia, diurnal variation, psychomotor retardation and agitation, neurocognitive deficits, severe physio-somatic symptoms, and mood-congruent delusions or hallucinations, severity of anxiety, overall severity of illness (e.g., as assessed with the Clinical Global Impression scale), and severity of suicidal ideation. The phenomenome is the aggregate of different aspects of the self-experience of illness, as assessed by using self-rating scales of depression, anxiety, health-related quality of life (HR-QoL), disabilities, trigger factors, and life events. Moreover, various symptomatome and phenomenome features should be combined into one latent vector which is the cause of all phenome indicators. For example, previous research [25] was able to extract one common core from CGI, suicidal ideation, severity of depression and anxiety ratings, the four domains of HR-QoL scores, and different domains of disability scores. The construction of such a latent variable outcome reflecting the phenome of severe depression contrasts with the status quo of psychiatry, which uses questionable DSM/ICD diagnostic groups or one folk psychology-derived rating scale as the outcome data [38,48].

Figure 4 illustrates a knowledge-based causal framework based on causal reasoning and current state-of-the-art research that describes the causal relationships between the various components of clinical depression. Expanding on this framework will require incorporating pan-omics data, environmental (lifestyle/nutrition/toxins) data, psychological (personality/coping mechanisms), social (socioeconomic/social support), and medical data (body mass index/comorbidities/heart rate variability) as well as socio-demographic data and other factors.

**Figure 5:**
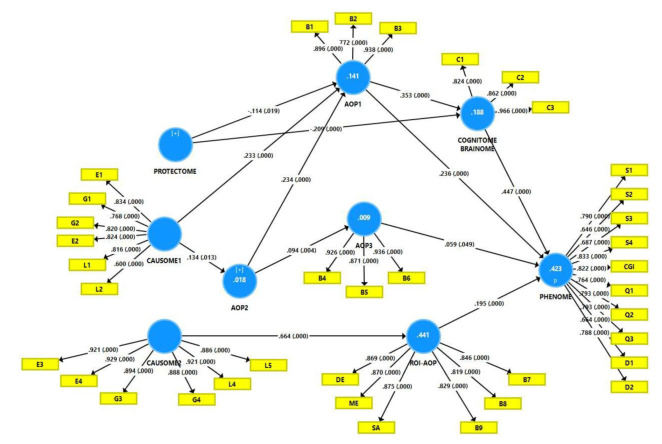
Example of a data-driven, bottom-up, digital model of depression in unipolar and bipolar disorder. This is an output of partial least squares (PLS) path analysis showing a multi-step mediation model linking risk-resilience factors (causome, protectome) with adverse outcome pathways (AOP), the reoccurrence of illness index (ROI), the cognitome-brainome, and the phenome of depression. G: genome markers; L: lifestyle factors; E: environmentome factors; B: biomarkers or biomarker sets; DE: number of depressive episodes; ME: number of (hypo)manic episodes; SA: number of suicidal attempts; S: symptoms and severity of illness scores, Q: quality of life data; D: disability data. ROI-AOP: a pathway phenotype, namely a common core underpinning ROI and neuro-oxidative pathways. Shown are path coefficients with exact p-value; white figures in the circles: explained variance.

Consequently, this framework may be validated using PLS path analysis, which enables the construction of latent vectors and the mapping of risk-resilience factors to AOPs, ROIs, and phenome constructs [25,26]. Figure 5 illustrates the output of a PLS analysis, which enabled the development of a new neuro-immune/neuro-oxidative model of clinical MDD/MDE that reassembled all components of depression into a data-driven, bottom-up, digital model that is cross-validated and proven to be replicable using the accurate statistical tests as described in [38]. It is important to note that this digital model allows one to assemble symptomatome, phenomenome and ROI features with multiple risk-resilience (genes, ELT, environmental, lifestyle) factors and multiple AOPs, thereby concretizing the abstract concept “phenome” as a neuro-immune and neuro-oxidative entity, a process dubbed “reification of the phenome of depression” [38]. As a result, rather than relying on precision psychiatry (a contradiction in terms), we constructed novel precision nomothetic models of clinical depression in our research.

**Figure 6:**
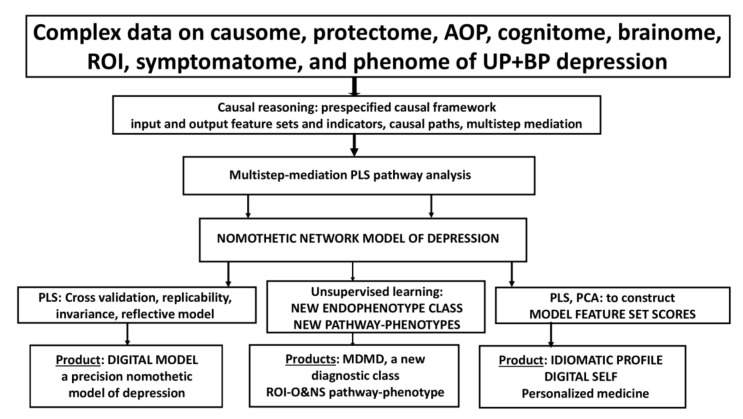
The major steps in precision medicine as applied in precision nomothetic psychiatry for constructing a new model of depression, new endophenotypes including major dysmood disorder (MDMD), and an idiomatic profile. ROI-O&NS: a pathway phenotype comprising staging of mood disorders and oxidative and nitrosative stress biomarkers.

**Figure 7:**
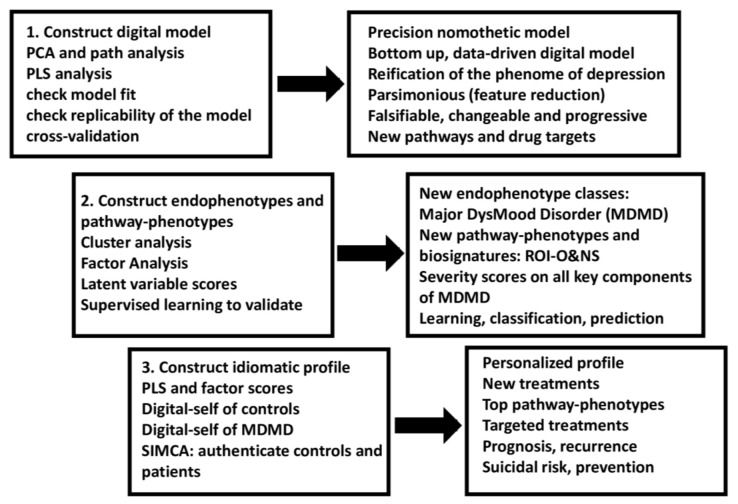
Three steps of precision medicine applied to precision nomothetic models of mood disorders and the different products generated during this process. These methods allow us to construct a digital giant model of depression comprising all building blocks of the disease, endophenotype classes (including MDMD or major dysmood disorder) and pathway phenotypes (e.g., ROI-AOP, which is a common core underpinning reoccurrence of illness and a specific adverse outcome pathway) and personalized scores on these new constructs and other indicators in the model which together delineate a idiomatic profile for each individual. ROI-O&NS: a pathway phenotype comprising staging of mood disorders and oxidative and nitrosative stress biomarkers.

Figure 6 shows the different steps in precision nomothetic medicine, from constructing a cross-validated model, extracting endophenotype classes and pathway phenotypes and the machine learning techniques that can be used to this end. Figure 7 shows the three steps of precision nomothetic modeling of mood disorders and the different products generated during this process.

This figure shows that partial least squares (PLS) pathway analysis may be used to compute a digital model of depression. Consequently, the new model is validated using various statistical techniques which check model fit, replicability, compositional invariance, and whether the factors are misspecified as a reflective model. Using unsupervised learning (e.g., various clustering techniques) new endophenotypes may be discovered. These new classes should be validated using supervised pattern recognition methods including soft independent modelling of class analogy (SIMCA), support vector machine (SVM), and neural networks (NN). The top features of the new endophenotypes should be determined using analysis of variance, SIMCA, and NN. Novel pathway phenotypes are constructed using PLS and principal component analysis (PCA). Indicator, latent vector, and pathway phenotype scores are computed to determine an idiomatic profile for each patient.

### 7.2. Construction of Endophenotype Classes and Pathway Phenotypes within the New MDMD Model

#### 7.2.1. New Endophenotype Classes

As discussed in the previous sections and shown in Figure 1, Figure 6 and Figure 7, the second step in the precision nomothetic process is to detect new endophenotype classes that cluster patients into meaningful subgroups and discover new pathway phenotypes in the model. Thus, after constructing the PLS depression model, latent variable scores of all constructs in the model may be computed, including different risk-resilience scores, different AOP scores, an ROI score, and cognitome, brainome, and phenome scores, which reflect the severity of the different building blocks of the disorder. Consequently, the scores are subjected to unsupervised learning, namely cluster analysis, to discover new subgroups of patients based on the features of the illness. Simeonova et al. [26] discovered that 70% of MDD patients were assigned to a novel cluster with increased phenome, bacterial translocation (leaky gut), and O&NS scores, indicating that our unsupervised machine learning disclosed a novel endophenotype class. Maes et al. [25] discovered that when PLS-derived scores for the key components were clustered in a sample of depressed patients (including MDD and MDE), a cluster characterized by increased risk-resilience, O&NS, ROI, and phenome scores was formed. Additionally, this new diagnostic class (MDMD) had a greater influence than the diagnoses of MDD or MDE or bipolar disorder, indicating that even these diagnostic categories are not important [25]. Therefore, we renamed the new endophenotype class with elevated risk-resilience, AOP, ROI and phenome scores “MDMD” and showed that this endophenotype class is due to increased neuro-affective toxicity. It is important to note that using SIMCA, we can construct digital (twin) models of the new endophenotype class MDMD, and a digital healthy model as well.

Our findings show that biological processes capture mental disease and suffering, that mental experiences are physically reflected, and that the phenome of mood disorders is predicted by biomarker combinations. Moreover, precision nomothetic psychiatry (Figure 6 and Figure 7) can be used to discover more influential diagnostic classes for mood disorders than those conceptualized in the current phenomenological classification (e.g., unipolar versus bipolar), and that this new classification should eventually replace the current ones after further validation and falsification. These findings refute Kohne and van Os’s contention that biomarkers are inadequate to capture mental experiences and that precision psychiatry cannot replace present phenomenological categorization methods [41].

Additionally, precision nomothetic models of mood disorders demonstrate that severe mental suffering may originate in the periphery, not necessarily the brain, implying that physical states (specifically activated neuro-oxidative and neuro-immune pathways) determine severe mental suffering in patients with mood disorders. This contrasts with the widespread folk psychology belief that “all is in the mind” [41], as well as with philosophy of mind and other philosophical-psychological mind theories, such as those on the immaterial mind, intentionality, neutral monism, and mental causation. MDMD is, in summary, a severe medical disorder with a distinct pathophysiology, namely neuro-affective toxicity, which has nothing to do with philosophy, qualia, or meaning.

#### 7.2.2. New Pathway-Phenotypes

Pathway phenotypes (biosignatures or endophenotypic quantitative scores) may be computed by extracting latent vector scores from highly interconnected paths, for example, linking ROI with cognitome and phenome features, yielding a ROI-phenome signature (Maes et al., staging index); linking ROI with ELT and cognitome data, yielding a causome-ROI-phenome signature [19]; or linking ROI with O&NS indicators, yielding a ROI-biosignature [57]. Such constructs are extremely important because they offer severity scores for specific pathway phenotypes and they allow one to learn from the data for example that the pathophysiology of ROI is tightly coupled with aberrations in O&NS pathways [57].

The ultimate goal of developing these digital, either new endophenotype classes or pathway-endophenotype scores, is to allow for more targeted interventions, either therapeutic or preventive, and to shorten the time for medical innovations, thereby reducing costs. As such, our results reveal that neuro-immune, autoimmune, O&NS pathways and lowered antioxidant defenses are new drug targets in MDMD and patients with an increased ROI.

#### 7.2.3. Towards Personalized Medicine

The last step in precision nomothetic medicine (see Figure 1, Figure 6 and Figure 7) is to develop a more personalized approach which will allow the individual patient to benefit from the construction of the nomothetic model, endophenotype classes and pathway phenotypes by predicting MDMD membership and treatment response, thereby opening the door toward preventive treatments. As previously stated, there are two primary statistical machine learning methods that can be used.

To begin, the latent variable scores derived from PLS, or factor analysis not only delineate the precision nomothetic model, the novel endophenotype class and pathway phenotype severity, but also build an idiomatic profile that is unique to each individual. Indeed, this unique profile incorporates risk-resilience, causome, protectome, multiple AOP, cognitome, brainome, symptomatome, and phenomenone scores, all of which contribute to the creation of a unique profile that varies between patients. Furthermore, the different feature set scores that comprise this idiomatic profile may show the top anomalies in causome, environmentome, AOP, cognitome, or brainome components in a certain person, allowing for the formulation of individualized treatment methods [38]. Applied to subjects at risk (those with symptomless illness), the model and pathways phenotype scores may reveal a propensity to develop MDMD (e.g., ELT combined with specific genes and increasing O&NS) or may predict the reoccurrence of MDMD (high ROI score) and thus suicidal behaviors, thus paving the way towards preventive medicine.

Recently, Al-Hakeim et al. [14,59] developed SIMCA as a useful machine learning method for predicting treatment response, and the same method may also be used to predict who is at risk for MDMD. In both cases, SIMCA PCA models surrounding normal volunteers are constructed using the model features (risk-resilience, AOP, ROI, cognitome, brainome and phenome) measured in healthy controls. SIMCA models of normal controls are composed of principal components extracted from the control pan-data and thus describe the feature similarities between those individuals [21,30]. Consequently, the SIMCA feature scores obtained in subjects at risk or in a clinical partial remitter may be projected into the class limits of the normal control model (see Figure 2), allowing us to authenticate subject as belonging to the normal control class or refute normal class membership. Thus, if a patient treated with antidepressant treatments or an individual at risk is not authenticated as a normal control (i.e., is not allocated to the SIMCA control class), the results indicate that the patient is not remitted (and thus is a partial or non-remitter) or is at risk to develop MDMD, respectively.

## 8. Conclusions

In this paper, we explain that precision medicine can be broken down into three steps: (1) optimizing and enhancing conventional models and generating digital twins; (2) using precision medicine to establish endophenotype classes and pathway phenotypes; and (3) developing a digital self for each patient. None of these phases have been fulfilled in psychiatric research. The failure of precision psychiatry to progress into actual sciences is due to the lack of an accurate (cross-validated and reliable) model of clinical depression as a severe medical illness distinct from an emotional reaction. We show how to use SIMCA, PLS path, factor, and cluster analysis to create a new precision nomothetic depression model.

The term “nomothetic psychiatry” refers to the ability to reify the clinical diagnosis of depression utilizing causome, protectome, cognitome, symptomatome, and phenomenome data, as well as AOP data. We use the term “precision” nomothetic psychiatry to describe how we employed precision medicine methodologies to uncover a new endophenotype class, MDMD, induced by neuro-oxidative toxicity, as well as new pathway phenotypes, such as ROI-AOP, which combines the ROI and O&NS pathways. It is worth noting that the machine learning approaches used in our research enabled us to create digital models of MDMD and controls (using SIMCA) as well as a digital self for a specific patient. Our endophenotype classes and pathway phenotypes (based on Thai, Brazilian, and Iraqi people) should be verified in separate samples, particularly in other Western nations, and should be optimized and enhanced using pan-omics and comprehensive brainome data.

## Figures and Tables

**Table 1:** Supervised and unsupervised pattern recognition methods useful in precision medicine.

	Supervised Learning	Unsupervised Learning
Definition	The use of labeled datasets to train algorithms capable of reliably classifying data or predicting outcomes. These data always consist of paired input (explanatory) and correct output data (dependent data, i.e., predefined classes, or continuous data). Training data are analyzed to produce an algorithm (model) that can be employed to map new cases for example as belonging to the depressed or control class. An algorithm is trained on the input data to detect underlying patterns that are associated with the prespecified output variables.	The computer algorithm learns from unlabeled datasets (training sets). The model determines the similarities or patterns in the input variables without associating these data with the output variables.
Examples	Support vector machine Neural networks Soft independent modelling of class analogy Linear discriminant analysis Multiple regression analysis Logistic regression analysis Decision trees Partial least squares path analysis	K-mean clustering K-median clustering Forgy’s clustering Hierarchical clustering Principal component analysis (PCA) + Correlation loadings + PC plot Exploratory factor analysis Correspondence analysis
Aims general	Classification Prediction of predefined classes or scale variables Mapping of unknown cases Learn from the data Delineating associations	Discovery of patterns Uncover clusters of cases Learn from the data Uncover associations between input variablesDelineating rules that describe the data
Aims precision medicine	Optimizing existing disease models Defining new pathways in the input variables associated with a disease Classifying unknown cases as a patient or control Cross-validation of new endophenotype classes Cross-validation of pathway phenotypes Construct new disease models based on causal associations	Construct new endophenotype classes Construct pathway phenotypes
Useful in precision nomothetic psychiatry	Partial least squares path analysis Support vector machine Neural networks Soft independent modelling of class analogy	Clustering techniques PCA PCA plot Exploratory factor analysis

**Table 2:** Building blocks included in the digital models of major dysmood disorder (DMDM).

Building Blocks Of Depression	Description	Examples in the Current Conceptual Analysis
Causome	All causal factors that increase risk toward MDMD (genetic, environmental, and lifestyle factors)	Early lifetime trauma (ELT)
Protectome	All factors that protect against the onset of MDMD (genetic, environmental, and lifestyle factors)	High high-density lipoprotein cholesterol paraoxonase 1 gene (PON1)
Risk-resilience index	Composite based on risk and resilience factors	Early lifetime trauma by PON1 gene interactions
AOP (adverse outcome pathways)	Pathways leading to a medical disease	Latent vectors extracted from neuro-oxidative and neuro-immune biomarkers
Brainome	Aggregate of brain imaging assessments	Changes in the brain connectome
Cognitome	Aggregate of impairments in cognitive functions	Latent vector extracted from executive, attention, and memory dysfunctions
Symptomatome	Aggregate of all symptoms, severity of illness, global clinical impression (CGI)	Latent vector extracted from symptoms, severity indices, GCI, suicidal behaviors
Phenomenome	Self-experience of the illness	Latent vector extracted from phenomenome data including self-rated disabilities and quality of life
Phenome	All symptomatome and phenomenome features	Latent vector extracted from symptomatome and phenomenome data

## Data Availability

Not applicable.
